# Youth perceptions toward managing elderly care among low-income household families using the My-Elderly-Care-Skills Module

**DOI:** 10.3389/fpubh.2023.1042124

**Published:** 2023-02-08

**Authors:** Nur Syuhada Mokhzan, Rosnah Sutan, Ruhizan Mohammad Yasin, Hamidah Yamat

**Affiliations:** ^1^Department of Community Health, Medical Faculty, University Kebangsaan Malaysia, Kuala Lumpur, Malaysia; ^2^STEM Enculturation Research Center, Faculty of Education, University Kebangsaan Malaysia, Bangi, Selangor, Malaysia

**Keywords:** youth, perception, elderly care, training, aged care

## Abstract

**Background:**

Caregivers of elderly people need the right education and empowering skills to manage their own health needs and the elderly people they care for.

**Objective:**

The study aimed to explore youth perceptions of the My-Elderly-Care-Skills Module intervention and its perceived feasibility.

**Methods:**

This study involved youth respondents (18–30 years old) from low-income households who are accountable to providing care for independent older people (60 years or above) living in the same house. A qualitative study using a case study design was used to assess youth perceptions based on the content of the My-Elderly-Care-Skills module, by focusing on its implementation usage and usefulness for the care of the elderly. A total of 30 youths voluntarily participated in the online training workshop during the COVID-19 pandemic movement restriction order period. There were multiple sources of data, such as video recorded on reflection of care given at home, text messages in a WhatsApp group, and in-depth interviews during small group online meetings. Data were recorded and transcribed verbatim for common themes before a theme analysis was conducted. Inductive content analysis was performed after the saturation point was met.

**Results:**

Thematic analysis derived two domains of feasibility: operational and technical feasibility. There were three themes under operational practicality (improving awareness, addressing the caregiving skills needs, and seeking resources for knowledge) and three themes for technical practicality (easily used and informative, skill in effective communication, and program fulfillment).

**Conclusion:**

It was verified that it is feasible for young caregivers of the elderly to participate in the My-Elderly-Care-Skills training intervention as it helps in improving knowledge and skills performance in managing and caring for the elderly.

## Introduction

Aging, health, and quality of life are closely intertwined. In Malaysia, the elderly are classified as those aged 60 or above (1). In 2020, the elderly population in Malaysia was 3.5 million, 10.7% of the total population, and this number is predicted to triple by 2040 when one in every five Malaysians will be aged 60 or above (2). The percentage of the elderly is projected to reach 15% of the total population in 2030, making Malaysia one of the most rapidly aging nations in the world (2). The current life expectancy for Malaysians is 72 years for men and 75 years for women (2), which is still far behind the life expectancy of 80 years in developed countries.

Elderly care and management strategies for promoting the wellbeing of both the elderly and their caregivers are still in the infancy stage in Malaysia. Most studies focused largely on the elderly and not on their caregivers (3). A recent systematic review assessing the gap in bridging health and social care of caregivers identified only 15 publications in Asia over the past 5 years that discussed the impact of interpersonal communication, social works intervention, and communication barriers faced by caregivers of the elderly (4). In addition, most studies concentrated mostly on awareness, education, and tasks of informal elderly caregiving in a familial setting, and scarcely focused on health problem-solving, dietary and nutritional requirements, and satisfaction with comfort, hygiene, daily activity, and safety of the elderly who lives alone or in the same home as the caregiver (4).

For statistical purposes, the Malaysian Youth Development Research Institute (IYRES) defined youth as the population between the ages of 15 and 30 (5). The numbers of the youth in Malaysia are as follows: 5,242,700 males and 4,709,900 females (5). The proportion of youth to the elderly is higher in Malaysia compared to the rest of Asian countries. As part of the sandwich generation and with an extended family living arrangement, most youths stay in the same house as their elderly relatives, either their own parent or a family member. Youths are the catalyst for the nation's future by spearheading the changes in the country's leadership. Their role in providing elderly care should be enhanced by better skills. They should also be better equipped to deal with the responsibilities of being part of the sandwich generation. Malaysia is transforming into a high-income nation through the Fourth Industrial Revolution (IR4.0), which demands a change in daily life, working style, communication, and how the future is evaluated (6). One of the most important aspects of IR4.0 is the realignment of the purpose of education, in that it should go beyond increasing knowledge to also help individuals understand how the learned information can be applied in daily life.

Based on the literature review of the evidence provided by previous studies, the researchers found that most of the studies only focused on the elderly respondents and not on their caregivers, especially in the case of young caregivers (7). Lack of awareness among the youth about the importance of elderly care was encountered and they were normally less interested in gerontology education (8). Lack of disclosure of care and the importance of taking care of elderly health was taught at universities, colleges, schools, and non-governmental organizations (NGOs). Poor awareness and scarce training make the youth less skilled in the care of the elderly and at the same time affects the quality of life, social support, and psychological wellbeing of the caregivers of the elderly, as well as elderly satisfaction.

Malaysia still lacks research on the various issues involved with professional care for the elderly, such as training modules for the elderly, and training of the skills needed by caregivers of the elderly and the disabled population. Most available skills were focused on hospitality skills, sewing, cooking, retail, and vocational training (9). Therefore, it is necessary to develop a specific module that promotes social participation in active aging and helps caregivers and the elderly to improve their quality of life, social support, and psychological wellbeing. The module should aim to empower youth to engage in training and vocational training (TVET) and to make them aware of the importance of empowering TVET in helping to improve the national economy and practicing lifelong education. Malaysia is still attempting to reach a high number of skilled workers to be comparable to developed countries, such as Taiwan, South Korea, Singapore, Germany, and the United Kingdom, which are some of the countries that consider TVET as one of the main focuses in education (10). A scoping review on informal caregivers recently reported that most training interventions helped in the caregivers' needs, specifically in improving knowledge, self-efficacy, and competency (11, 12). A qualitative approach study among the caregivers of the elderly mostly reported on their physical and mental readiness, and knowledge related to elderly care and support (4, 13). There is limited literature on exploring perceptions, especially among the youth in caring for the elderly. Therefore, the present study aims to explore youth perceptions of caring for their elderly relatives concerning the training intervention modules given to them and their perceived feasibility.

## Methodology

The study employed the case study as a type of qualitative research to explore youth perceptions of using the My-Elderly-Care-Skills module online training in caring for the elderly in relation to the training intervention modules given to them. Using the qualitative case study methodology enables researchers to conduct an in-depth exploration of intricate phenomena within some specific context (14, 15). The study was conducted during the Malaysia Government Movement Control Order (MCO) at a time when several phases had been enacted nationwide to curb the COVID-19 pandemic and had put various sectors of the economy in jeopardy (16). The MCO caused many activities reliant on services to allow people to work and learn from home through the utilization of online platforms. We adopted an e-learning technique to teach the My-Elderly-Care-Skills module and to train youth in caring for their elderly relatives at home during the MCO. We aimed to provide accredited training based on the module developed and tested among the youth as on-the-job training and to provide a support system to help youths secure jobs in elderly care in the community or institutional care. The case study approach is particularly useful to employ when there is a need to obtain an in-depth appreciation of an issue, event, or phenomenon of interest, in its natural real-life context (15).

We reported the qualitative research output following the suggested guideline (17, 18) and added it in the Supplementary file 1 for reference. Each focus group discussion consisted of five respondents and each session lasted approximately one to one and a half hours for each group, as described in the Supplementary file 2. Each respondent was used as case study data. The My-Elderly-Care-Skills module was created by adopting elderly care guidelines developed (19, 20) for educating the elderly and their caregivers on the various aspects of caregiving needs for the elderly. The research team used the guidelines and developed online presentations, interactive lectures, videos on caregiving skills, frequently asked questions, and tips for quick assessment. The module was divided into two set packages, i.e., the education package and the competency skills in managing the elderly package. The education package was divided into four domains: (i) the right of youth to obtain health information for self-care; (ii) healthy lifestyle approaches for wellbeing and active aging; (iii) preventing abuse and mental health resilience; and (iv) social support approach toward healthy aging. The competency skill in the elderly health care management package was divided into four domains: (i) youth role as an elderly healthcare volunteer; (ii) physical and mental health care for the elderly; (iii) tips to prioritize the needs of the elderly and seeking additional resources; and (iv) elderly health monitoring plan for the volunteers. These packages were designed to provide basic guidelines for providing care to the elderly and enhancing the competency skills of the caregivers. Researchers used the Google Meet application instead of a face-to-face workshop from March to October 2021 (during the COVID-19 pandemic's critical phase). Young caregivers of the elderly from all over Malaysia were invited to attend an elderly health empowerment webinar. The workshop was advertised *via* the Community Health Department, Faculty of Medicine University Kebangsaan Malaysia website in collaboration with the National Elderly Development Association (NEDA Malaysia.org), which is a registered non-profit organization, as well as the university's various official social media accounts.

### Setting and participants

The study was conducted during the height of the COVID-19 pandemic under the movement and quarantine restrictions imposed by the Malaysian Government's Movement Control Order (MCO) in 2020 and 2021. A case study design was used to explore the youth's perception of using the My-Elderly-Care-Skills module and its feasibility as a guide for improving caregivers' competency and feasibility analysis (technical and operational feasibility) for an assessment of the practicality of using the module. Each youth who participated in the training was invited as a case study respondent. Respondents were the youth who is the main caregiver of the elderly and living in the same home as the elderly relative. The selection was based on the respondent's ability to participate in the two-day My-Elderly-Care-Skills Module workshops and their ability to provide sufficient data on their experience and the effectiveness of the module in empowering their competency skills. The inclusion criteria were: Malaysian citizens who can speak and understand Malay or English, have access to a smart device with a stable internet connection, age between 18 and 30 years, are from low-income household families (< RM4,850 (USD1050) per month or known as B40 category) (1, 2), and are caregivers to a dependent elderly person(s) staying together in same living arrangement. All respondents consented to participate in the study. We selected the youth who used the My-Elderly-Care-Skills module for 3 months and completed the 2-day online training course. There were 30 youths selected purposively to give a response to a key question during our data collection period.

### Study instrument

The respondents were interviewed at the end of the intervention using the online platform (Google Meet application) for ~5–15 min each in an individual in-depth interview using an online focus group discussion (FGD) technique. The groups were formed based on the respondents' residential state: north, south, central east, and west of the Malay Peninsula to ensure they had the same language dialect and social-cultural background. Monthly monitoring was also conducted *via* video calls, video recordings, and WhatsApp conversations over a period of 3 months since its implementation. The qualitative method in the study used the lived experience of the youth to assess the experience of young caregivers in caring for their elderly relatives and thematic analysis was performed to interpret the data to answer the study's objective. The questions were constructed as semi-structured, i.e., open-ended and closed questions.

- How do you find the My-Elderly-Care-Skills module and training in helping your understanding and skills in improving healthcare for the elderly?
- What did you learn after attending the training webinar over the past 2 days?
- Did this training webinar help you in caring for the elderly?
- What challenges do you face in using the information given in the module?

### Data collection tools

WhatsApp messages, recorded videos, and Google Meet recordings during the interview were transcribed and used in the present study. Table 1 listed the tool used and its function. Each respondent needed to have a training module (e-book), and a computer with internet access. The researcher (RS) provided online training on the use of the module and acted as the facilitator. Another researcher (NSM) acted as a moderator during the training and also conducted the focus group discussions as well as transcribed them verbatim.

**Table 1:** List of materials used and the roles of the researchers and respondents.

	**Researchers**	
**Respondents**	**Facilitator (RS)**	**Moderator (NSM)**
1. My-Elderly-Care-Skills module 2. Laptop/desktop computer 3. Good Internet access	1. Laptop/desktop computer 2. Good Internet access 3. My-Elderly-Care-Skills module presentation, lecture note, video, and questionnaire to be given to trainees 4. planned activities for the program to be carried out.	1. My-Elderly-Care-Skills module 2. Laptop/desktop computer 3. Good Internet access 4. Lead the training program 5. Prepare poster slides to be posted during the training program.

#### WhatsApp

- WhatsApp is a free messaging platform that sends and receives texts, images, audio, and video files as well as web addresses to and from individuals and groups; it also served as an important e-learning platform. The researchers formed a WhatsApp group for the “Caregiving Training and Education for the Elderly Workshop” to facilitate communication of the My-Elderly-Care-Skills module usage and sharing experiences. NSM compiled all the respondents' reflection feedback, acknowledged their contribution, and introduced them to one another for sharing their experiences and opinions in caring for the elderly.

#### Video recording

- Participants were requested to produce a short video while caring for the elderly for the researchers to assess their feedback experience in the implementation of the module for the daily task of caring for their elderly relatives at home.

#### Google Meet

- Google Meet was selected as the medium for focus group interviews as it is a simple and free platform. Participants were requested to enter the Google Meet room according to the schedule provided by the researchers. Everyone was allocated ~5–15 min to reflect on their experience and the challenges faced.

### Data collection procedure

Data were collected in three phases over a period of 4 weeks. At the start of phase one, informed consent was obtained from the respondents, and they were briefed on the purpose and duration of the study before proceeding to the interview session. Focus group discussion interviews were conducted for an average of an hour to an hour and a half with five respondents at each time during phase two, which was conducted over 6 days (4–9 October 2021). During each FGD, everyone was given ~5–15 min to voice their opinions. Six focus group discussions were done with 30 respondents. Each respondent's view was treated as a case study. Each session was recorded and transcribed for data recording purposes. At the end of 3 months, we asked the respondents to submit a short video with their reflection about the training and usage of the module.

### Data analysis methods

Data analysis was performed after verbatim recordings had been transcribed. Researchers constructed three boxes that were divided into themes, subthemes, and verbatim outcomes; following which, keywords collected in the verbatim outcomes were collected as themes. It is a form of pattern recognition used in analysis whereby themes that emerge from the data become the categories for analysis. The process involves the identification of themes with relevance specific to the research focus, the research question, and the research context that allows data to be both described and interpreted for meaning (21). All researchers worked together to explore the verbatim transcripts and reached a consensus for thematic analysis based on the feasibility component: technical and operational practicality (Table 2).

**Table 2:** Flow of thematic data analysis.

**Step 1**	**Step 2**	**Step 3**
**Read and underline keywords**	**List the underlined keywords**	**Unify the obtained keywords by forming a theme**
**Example of verbatim recording**	**Subtheme**	**Theme**
…all the information that I got, the youth need to take care of five main things in the care of the elderly. Among them are comfort, food, cleanliness, daily activities, and safety... (R3)	Acquired knowledge through training	Knowledge Enhancement (operational practicality)
...applying the five main focuses in planning for the elderly to my mum. [*sic*] The five main focuses are in terms of comfort, food, cleanliness, daily activities, and safety at home...(R7)		
...I need to take care of the quality and quantity of his food and ensure safety i.e. no small toys for his grandson along his journey because he is worried about being stepped on and so on.... (R3)	Addressing the caregiving skills needs	The benefit of elderly care (operational practicality)
...I am more aware and know the importance of taking care of the emotional and mental health of the elderly...(R13)	Improve awareness	Awareness (operational practicality)
...this program is also very good because it cares about the mental and psychological health of the elderly... (R10)	i. Easy to use and informative. ii. Skills in effective communication. iii. Program fulfillment.	Program Completion (technical practicality)

### Validity and reliability

**Figure 1:**
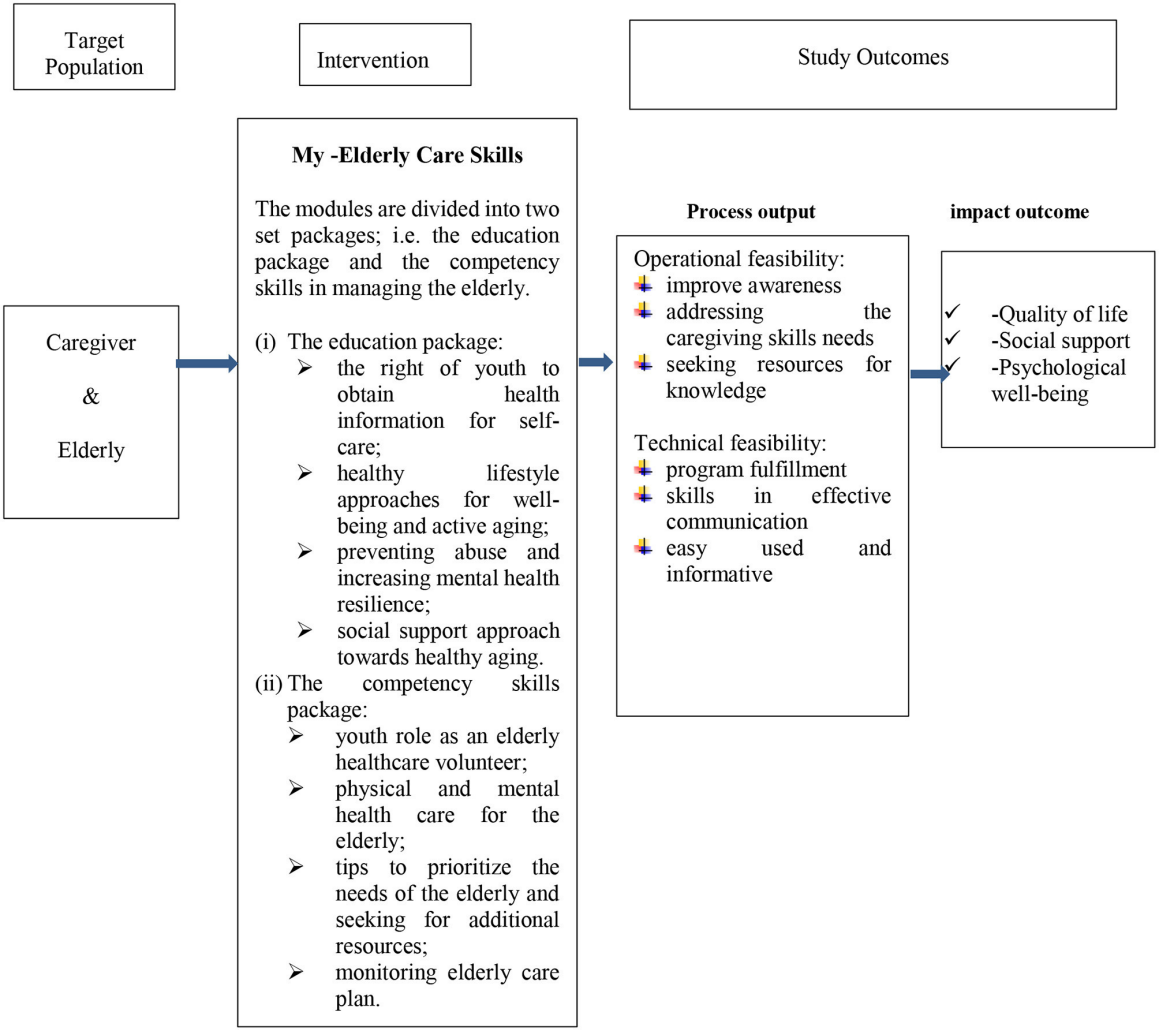
Visualization of study framework and output.

All six FGD interviews were moderated by NSM and assisted by RS. To ensure the validity of the research, data from the FGD (video interviews using Google Meet), WhatsApp group chats, and reflection videos on the module's feasibility, were used as a triangulation approach to achieve consistency and uniformity to answer the research question. The study set a primary research question as to how the youth perceived caring for their elderly relatives by using information gained during the online training of the My-Elderly-Care-Skills modules. The key questions were developed by the research team as listed in the Supplementary file 3. They were listed to address our concern on feasibility assessment that was not covered in the quantitative study design questionnaire. In exploring the respondents' perception of using the module, we related the key question with the content of the module as summarized in Figure 1. In assessing for reliability, we distributed the verbatim transcripts among the other researchers (RMY and HY) who were not involved in the interviews to find similar patterns. The interview questions and thematic analysis techniques were cross-referenced with experts (HY and RMY) who have experiences of over 25 years of qualitative study design for validation before and after the interviews were conducted. To ascertain reliability, reduce misinterpretation on the researchers' part, and fill in data gaps, the transcriptions of the interviews were shared with the respondents for them to review and a follow-up discussion was conducted to determine if the researchers understood them correctly.

### Research findings and discussion

This section evaluated the findings of the interviews based on the 30 respondents who participated. The result was arranged to answer the study objective, which was to explore youth perception of the My-Elderly-Care-Skills Module intervention and its perceived feasibility. Demographic details of the respondents are outlined in Table 3.

**Table 3:** Respondents' profiles.

**ID**	**Age**	**Gender**	**Ethnicity**	**Age of elderly in their care (years old)**	**Elderly health status**		**Relationship with the elderly in their care**	**Length of the period for care for the elderly (hours per day)**
					Needs moderate daily care monitoring (has multiple chronic illnesses that need to comply with treatment)	Needs basic care in monitoring (has a mild illness and can manage themselves)		
R1	24	Female	Malay	63	**/**		Child	3
R2	24	Female	Malay	80		**/**	Child	3
R3	20	Female	Malay	72	**/**		Child	3
R4	24	Female	Malay	60		**/**	Grandchild	3
R5	24	Female	Malay	83		**/**	Child	3
R6	24	Female	Malay	78	**/**		Grandchild	3
R7	21	Female	Malay	81		**/**	Grandchild	3
R8	23	Female	Chinese	65	**/**		Grandchild	3
R9	24	Female	Malay	74		**/**	Child	3
R10	24	Female	Malay	63	**/**		Grandchild	3
R11	22	Female	Malay	80		**/**	Child	3
R12	24	Female	Malay	74	**/**		Grandchild	4
R13	20	Female	Malay	63	**/**		Grandchild	3
R14	26	Male	Malay	70		**/**	Grandchild	3
R15	25	Female	Malay	73		**/**	Grandchild	3
R16	24	Female	Malay	60	**/**		Grandchild	3
R17	21	Female	Malay	77		**/**	Child	5
R18	23	Male	Malay	63	**/**		Grandchild	5
R19	29	Female	Malay	68	**/**		Grandchild	3
R20	24	Female	Malay	65	**/**		Grandchild	3
R21	19	Male	Malay	60		**/**	Grandchild	4
R22	23	Female	Malay	67		**/**	Child	3
R23	24	Female	Malay	67	**/**		Grandchild	3
R24	27	Female	Malay	61		**/**	Grandchild	4
R25	25	Male	Malay	63	**/**		Grandchild	3
R26	27	Female	Indian	62	**/**		Grandchild	5
R27	27	Female	Malay	61	**/**		Grandchild	4
R28	25	Male	Malay	60	**/**		Grandchild	4
R29	23	Female	Malay	78	**/**		Grandchild	4
R30	24	Female	Malay	78	**/**		Child	4

### Respondents' profiles

The age of the respondents ranged from 19 to 29 years. The majority were Malays (*n* = 28/30), women (*n* = 25/30), and grandchildren (*n* = 21/30). Most of them cared for their elderly relative, whose ages ranged from 60 to 81 years old. The majority needed moderate care monitoring as the elderly suffered multiple illnesses that required proper treatment compliance to drugs, physiotherapists, clean wounds, and clinic follow-up. Most caregivers spent ~1–3 hours per day managing elderly care needs. Approximately three-quarters (22/30) of respondents of the study were grandchildren.

### Thematic analysis

We assessed the youth's perception of the feasibility of using the My-Elderly-Care-Skills module in managing their elderly relative at home. Referring to Table 2, our finding achieved three themes under operational practicality and three themes for technical practicality. We reached the point of saturation after the fourth FGD and continued to proceed until all six FGDs were completed. Our approach was based on inductive understanding focusing on the perception of the module's feasibility in the care of the elderly. Table 4 depicts the quotes representing the themes for two domains of feasibility: operational and technical feasibility. Three themes under operational practicality were grouped as improving awareness, addressing the caregiving skills needs, and seeking resources for knowledge. Another three themes for technical practicality were categorized as easily used and informative, skill in effective communication, and program fulfillment.

**Table 4:** Summary of the results for theme development in relation to the feasibility domain.

**Feasibility domain**	**Themes**	**Quotes**
Technical feasibility	Easily used and informative	“*I used inputs given to me which is very clear to follow, and I am satisfied …”(R20) [sic]* “*1 learned that skills in caregiving for the elderly in the present era are very challenging and need the training to catch up with our skills. (R23)” [sic]* “*I learned so many new things in how the best caregiving methods for the elderly that tailored to my cultural beliefs…”(R18) [sic]* “*…so many things I learned during the workshop. I was never exposed to or learned while I was in school. In other words, it was easy and valuable new knowledge for me...”(R21)* “*…gave me new insights on so many new information on how to best care for the elderly and the relevant techniques …“ (R22)* “*… gained so much valuable information on managing and caregiving for the elderly...”(R25)* “*I gained a lot of new information on how to care for the elderly. I now know better about the aging process, and the tips on health evaluation, and how to plan caregiving for the elderly has been most helpful …”(R7)* “*I gained a lot of new information on how to manage caregiving for the elderly, especially on the health, physical, and mental care aspects … ”(R10)*
	Skills in effective communication	“*I have been exposed to the proper way of caring for the elderly, particularly on the best way to communicate with people who are older than me. It helped me gain new knowledge to prepare myself to care for and treat the elderly … ”(R4)* “*I gained more knowledge on managing and caregiving for the elderly. I learned how I can communicate better, be more mindful of their emotions and feelings …” (R6)* “*I gained exposure to the right and proper ways on how to be a caregiver for the elderly...”(R2)* “*I learned how to communicate more effectively with the elderly …”(R28)* “*I learned how I can communicate better with them and be more mindful of their feelings and emotions … ”(R29)* “*I learned about the techniques in caring for the elderly to be more effective with proper application on the physical and mental aspects from the training …” (R11) [sic]* “*the moderator and facilitator provided good explanations and in-depth exposure to the role of youth in caregiving. I also appreciate the sharing experiences by other caregivers of the elderly …” (R12) [sic]* “*I learned and helped me to add my knowledge on caring for the health of the elderly either on the physical or mental aspects … ”(R27) [sic]*
	Program fulfillment	“*… this is a wonderful program that emphasized the importance of caring for the elderly”s mental and psychological health … the program should be expanded to the wider community so that the physical and mental health of the elderly in this country are better-taken care of by the people who love them the most …” (R9)* “*... the program is a proactive measure in educating the community on proper caregiving for the elderly …” (R16)* “*… the program has a positive impact on the community, especially for youths like myself …” (R23)* “*… an effective way to educate the community on proper caregiving for the elderly …” (R25)* “*… understand some of the main focus when planning healthcare for the elderly, such as comfort, diet, hygiene, daily activities, and safety in the home to fulfill the needs of the elderly as best as possible …” (R10) [sic]*
Operational feasibility	Acquired knowledge through training	“*I gained new knowledge on caregiving for the elderly on aspects, such as the physical, emotional, and spiritual...” (R1)* “*the training exposed me to the physical and mental changes experienced by the elderly which led to their health problems …”(R18) [sic]* “*I can understand better now what should be my role as a child who will care for my father”s mental and physical health later on … the tips on evaluating my father”s health will help me plan on how I can be the best caregiver for my father …”(R24)* “*… the best knowledge I gained is on the five main factors I must pay attention to while being a caregiver to my parents. That is my parents” comfort, nutrition, hygiene, daily activities, and safety. (R3) [sic]* “*Now I will apply the five main focuses in planning for elderly caregiving for my mother. The five factors are comfort, nutrition, hygiene, daily activities, and safety at home. (R7)* “*This is my first time exposed to the best practices in managing and caring for the elderly as well as the actions that I can take when I am stressed while caring for my ill grandparent …” (R26)* “*I gained tremendous valuable input and information on caregiving for the elderly, especially for those with health and mobility issues like my father who has diabetes and had suffered from stroke…” [sic]* “*I gained so many valuable inputs like providing right and consistent attention while being a caregiver to the elderly …” (R8)* “*I obtained so many inputs on how to manage emotions, communicating techniques and so on …” (R15)* “*I find that many inputs and information on the best practices to care for the elderly …” (R9)*
	Improve awareness	“*… I am more aware and mindful of the emotional and mental care needed by the elderly …” (R13)* “*… to pass on the awareness to the greater community as a caregiver to the elderly …” (R18)* “...*modules we have just seen that there are actually many elements that youth need in caring for the elderly, among them in terms of emotional, physical, and mental health because the elderly*...” (R14) “*… it made me more mindful of our responsibility as a caregiver …” (R18) [sic]* “*… to be more attentive, such as giving their medication according to the prescribed time to reduce their discomfort …” (R8)* “*… I realized the importance of managing emotions and better communication approach …” (R5) [sic]* “*… to prepare me physically, emotionally, and mentally when dealing with their fussiness, grouchiness, and reclusiveness …” (R10)* “*… gave me early exposure on how to get further information to prepare myself to be a caregiver to the elderly in the future...” (R17)* “*… equipped with the knowledge needed to care for the elderly so we can take care of our parents and grandparents …” (R19)*
	Addressing the caregiving skills needs	“*I must take care of the quality and quantity of their food, making sure that none of the grandchildren's toys are in the way that can trip them or hurt them in any way...” (R3)* “*I must ensure that their daily routine, such as wound cleaning and dressing at the health clinic, is on time and to include light physical activities” (R3)* “*… to monitor my father's present health status and ensure that he is taking the medications as instructed by the hospital doctor …” (R13)*
		“*I will be more focused on their mental and emotional health” (R13)* “*I realized that I need to take a bigger role in taking care of their emotional needs so that they are comfortable and not offended …” (R14)* “*I am feeling more comfortable and still can help to relief my father's aches and pains after doing such physically demanding jobs …” (R24) [sic]* “*the training guided me on how to help to be the better person facing with problem-solving while taking care of my Grandma …” (R20) [sic]* “*… to create a daily schedule so that their day-to-day activities go smoothly. It would also help me monitor the medication schedules better …” (R29)* “* I apply what I learned at the workshop and it helps me to reduce the conflicts that I have with my mother. Now I understand her better and how loneliness from getting older affects her …” (R28)* “*… better equipped to deal with their moods because older people to be quite sensitive, some of the things we say can easily hurt their feelings …” (R30) [sic]* “*… can reduce the burden of caregiving once we know the right techniques in caring for the elderly …” (R19)* “*… helped me to manage the elderly in my household better and more efficiently …” (R25)*

#### Technical feasibility: Easily used and informative

Respondents provided positive feedback of the workshop. The workshop was described as clear for them to understand and follow step-by-step even though they had not previously learned about the subject in school. They attempted to try the techniques provided in the module at home while managing their elderly relatives. Respondents stated that the knowledge they gained from the workshop was beneficial and they were able to apply the provided healthcare management tips in their daily activities.

#### Technical feasibility: Skills in effective communication

Respondents stated that the training workshop was invaluable in their gaining new knowledge on caregiving as well as planning the care and treatment for the elderly, particularly on the communication aspect. They felt that they were better equipped to manage the elderly in their care, particularly from the communication and emotional need aspects. The workshop training, delivered as an online platform, helped them to understand all aspects a caregiver needs in order to improve caregiving quality and better fulfill the needs of the elderly relative with multiple co-morbidities who is in their care. The majority of the respondents indicated that the workshop helped to increase their knowledge and technical skills in caregiving from the physical and mental aspects, as well as their role as caregivers.

#### Technical feasibility: Program fulfillment

The majority of the respondents agreed that the training module provided good educational materials and taught them how to obtain more information on the best practices to manage and care for the elderly.

#### Operational feasibility: Acquired knowledge through training

The majority agreed that they gained new knowledge on caregiving for the elderly in terms of physical, mental, and healthcare requirements as well as tips on evaluating the health status of the elderly. The respondents gained additional knowledge on elderly care and management needs for illness monitoring, which improved their caregiving planning. A proper time plan guide helped them to improve their wellbeing. Awareness of the benefit of proper time management and understanding the specific needs of the elderly with illnesses boosted the caregiver's confidence and continuity of care for the elderly. The finding noted that the My-Elderly-Care-Skills module was well accepted by all respondents and provided important knowledge in caregiving for the elderly. They acknowledged that the training helped them to improve the quality of the caregiving they provide for their elderly relatives.

#### Operational feasibility: Improve awareness

Respondents reported that they felt that as caregivers to the elderly they needed to learn how to better manage their emotions and improve their communication skills. They also reported that they must be prepared to address the physical, emotional, and mental health needs of the elderly while dealing with their fussy whims, cantankerousness, and reclusiveness. They claimed that the module and the training helped them realize the importance of early exposure to elderly caregiving so that they could be prepared to fulfill their caregiving responsibilities. According to the respondents, the module helped them to become more conscious of their responsibility in caring for elderly emotional, physical, and mental health as well as their healthcare needs. They claimed to have felt empowered to share their knowledge and experiences within their communities.

#### Operational feasibility: Addressing the caregiving skills needs

All respondents claimed to be aware that caregiving for the elderly is not an easy task. They described it as requiring responsibility, trust and being a task that must be taken seriously by the caregivers. They claimed to feel more prepared to deal with the emotions and behaviors of the elderly in their care after the workshop. They reported feeling more equipped as a caregiver to manage and care for the elderly after the workshop by using the techniques they learned, especially in terms of quality of care.

## Discussion

In assessing the perceptions that the youth have on how to manage the elderly they care for in their homes, it became clear that more efforts are needed to empower young caregivers by providing them with the flexibility of ready-to-use materials, especially when there is limited access to formal training or institutional centers. Even though Malaysia is an aging nation, proper training for skill empowerment is limited and expensive, making it difficult for the country to prepare for an efficient treatment of its aging population, especially during disasters and economic downturns. In general, elderly care education and training is often a globally discussed and debated topic as the world faces an increased demand for the care of elderly populations (20). The creation of the My-Elderly-care Skills module is one of the steps that can be taken in helping the community to increase its knowledge about the care and health of the elderly while being able to achieve the government's desire to nurture the community, especially in helping the youth of low-income families practice lifelong education.

The results of the study showed that after attending the My-Elderly-care Skills workshop, the respondents were able to increase their knowledge related to the care and management of elderly health and were able to better plan the elderly care needs for the sake of mutual wellbeing. Knowledge is the main aspect of life to guarantee quality of life. In a recent national study, which used quantitative methods, with 1,153 Malay respondents aged 40–59 years, it was found that the quality of life among the elderly is very dependent on the caregivers of the elderly and that knowledge is the main aspect in helping the caregivers provide the best care services (22). Quality of life has a relationship with the increase or decrease in wellbeing in the life of caregivers and the elderly (23, 24).

Our present study explored the perceptions of young caregivers for the elderly during the COVID-19 pandemic. To present we have not seen any publication providing online training on the care of the elderly in the Asian region. Feedback from the respondents who participated showed a potential platform for training the younger generation and how they can use an online platform that focuses on elderly care. As many countries are facing the aging phenomenon, enhancing digital education will facilitate disseminating skills training for all in need. Our neighbor country Thailand shares almost similar socio-cultural practices and has developed a social health care program for the elderly in the community known as the Excellent Happy Home Ward (EHHW), which was carried out in the Lopburi Province, Thailand (25). The EHHW program involves taking care of the physical and mental health of the elderly and empowering the elderly in the use of information technology to help improve their support system and at the same time increase the level of knowledge of the elderly and caregivers of the elderly in the community. However, it required the caregiver to go to the center to obtain the training rather than be at home while managing their elderly relative.

Our findings showed that the benefit of the module is that it can be used for empowering caregivers in providing care for the elderly. Many researchers believe that the current trend of caring for the elderly depends a lot on young people who are not married, and it can also be seen that women are often chosen as caregivers for the elderly (22, 23). Our finding supports earlier evidence that women are more involved in elderly care. The elderly also often expect help and support from daughters because they are seen as easy to communicate with and easy to ask for help compared to sons who are seen as busy with work and have to shoulder multiple responsibilities, such as being responsible as the head of the family, as a father, as an employee, as a husband and as a child to their elderly parents (22). This phenomenon can have implications for the career, family, and national development of women who become tasked with various responsibilities. Indirectly it will have an impact on the wellbeing of the lives of the youth and the elderly (23, 24).

In this study, the researchers also found that six respondents stated that their role as caregivers of the elderly was not just living with the elderly as a companion, but the care of the elderly included emotional care, physical care, managing medication, ensuring mental and emotional health, as well as managingtheir own health status as they realized that they are the ones responsible for taking care of and managing elderly wellbeing. Our findings supported the perception of various ethnic groups that children and family members play a very important role in elderly caregiving and that children need to have a responsible attitude to repay the sacrifices of parents. There was also the notion that they too will also become old at some later point and maybe need caregiving from their own children (26, 27). A recent study also reported that the caregivers acknowledged their parents had sacrificed a lot to raise children–their children (26). It was clear that young caregivers of disabled elderly need family development support to make them emotionally and physically balanced, and need assistance in their interpersonal care so that they are ready to assume the role and responsibility of caregiving. Emotional care is key for the mutual wellbeing of the caregiver and the elderly under their care (28).

The results of the thematic analysis also showed that awareness among caregivers of the elderly was one of the priority needs. A previous study conducted on five senior respondents aged 60 or over, seniors living alone, and seniors experiencing physical, emotional, as well as financial, and material neglect in Negeri Sembilan and Selangor found that the communication gap between the caregivers and the elderly is caused by the lack of awareness of their role as caregivers of the elderly. There is no doubt that those caring for the elderly go through various challenges, including the changing nature of the elderly, who need love, attention, and friendliness from caregivers and the people living around them to achieve wellbeing in their daily lives (29). Our study found that nine out of 30 respondents indicated that the workshop was instrumentally able to increase their awareness of responsibilities as caregivers to their elderly. A previous study among the Malays also found that people who chose to be caregivers to the elderly tended to have a strong sense of responsibility and empathy (26).

Japan, Sweden, China, and Singapore are developed countries that have been facing elderly problems earlier than Malaysia, and they reported that the elderly are never left out of their country's various levels of planning, and that establishing close relationships with children, families, and caregivers of the elderly has always been a priority (3, 7, 13, 20, 26). Promoting visiting each other often, calling when living far away, and helping elderly people from nearby neighborhoods living in their community helped in enhancing community participation (30). However, Malaysia is facing rapid aging as it has progressed from an agricultural to an industrialized country that urges the migration of people to urban areas, which in turn leads the young population to live with family. The focus and spiritual attitudes of children, family members, communities, and guardians are shifting from looking after the welfare of the elderly. This coupled with the rate of economic improvement, lack of jobs, and the ever-increasing pressures of life have caused many communities to be less concerned about the problems of others, especially the elderly (31).

Our study aimed at improving knowledge and skills in the care of the elderly, and we hope it will open job opportunities for youth in providing elderly care services at an institutional-based level, home care or in visiting services once they are competent after attending the training and using the My Elderly-care Skills module. The module motivates the youth and the elderly to stay healthy and ensures quality of life, social support, and mental health wellbeing. It uses a digital platform that attracts engagement in the empowerment of caregivers in venturing into Vocational Education and Training (TVET) and does not focus on education alone. The present study involved nine freshly graduated students who showed interest for lifelong learning even with simple 2 days of online training based on the My Elderly-care Skills module. This finding supported intergenerational relationships and psychological and social support in the care of the elderly as presented by a case study in Kota Kinabalu. That study found the need to build a holistic module based on the National Elderly Policy (DWEN) and that there is a need for improvements in the national action plan so that the production of modules is more integrated to guarantee the wellbeing and quality of life of caregivers and the elderly (32).

The My-Elderly-Care Skills module was created with a proof of concept to raise awareness on the importance of elderly caregiving. It also imparted skills and knowledge to caregivers of the elderly and improved the wellbeing of caregivers to the elderly from the quality of life, social support, and psychological aspects. The module was constructed based on the social health intervention theory (33) that is divided into role boundaries, the burden of demand, and conflicts as part of the experience of a caregiver for the elderly that has to undertake various responsibilities simultaneously (i.e., as an employee, spouse, parent, child, student, and caregiver) in improving the quality of life, social support and wellbeing of both the elderly and their caregivers.

Thus, the My-Elderly-Care Skills module can be used to promote reciprocity between the elderly and their caregivers. According to the social health intervention theory, the wellbeing of the elderly and their young caregivers depends upon their quality of life, social support, and psychological wellbeing, with the latter two affecting the first (24, 34, 35). The elderly and their caregivers who received social support had a high level of psychological wellbeing and were less stressed. Thus, good social support will increase the psychological wellbeing and quality of life of both the elderly and their caregivers in the long run. Using digital platforms as media for training helped in bigger-scale participation for awareness and knowledge dissemination. However, competency skills are more appreciated with physical interaction training (36). The emotional aspect of caregiving addressed by the respondents in this study, such as fussiness, irritability, and reclusiveness, need to be prioritized in areas of problem-solving ability, self-care, and resilience against life's pressure and demands. Young caregivers and the elderly who are less stressed are in a better place emotionally when compared to those who are struggling under the burden of various responsibilities and life's demands that affect their psychological wellbeing. The youth of the 21st century fall under the sandwich generation that needs to bear various responsibilities simultaneously. The style of caring for the elderly at this point is not the same as before due to the phenomenon of changes in terms of family structure, modernization, and urbanization. Therefore, the caregivers of the elderly need help and support from formal or informal institutions to correctly manage the care of elderly relatives and not put any pressure on them in achieving better wellbeing in their families. As more of them have to take up the role of caregivers to the elderly, they must be equipped with the best knowledge and skills in preparation for Malaysia's aging phenomenon, which is due to start in approximately 2030. In the present study, we used multiple approaches based on the time and setting of a similar case study. Using multiple case studies approach using a depth case study helped in improving saturation and validity (14, 15).

## Limitation

The study had a limitation in exploring the perception experience in the aspect of technical feasibility related to environmental support. The study was conducted online, and the researchers had no responsibility for the respondent's internet connection at home. All respondents described caregiving activities based on their home setting. The study was conducted during the critical phase of the COVID-19 pandemic where the majority of places were imposed with a movement restriction order (MCO). The majority of the youth who participated in the study were trapped as the caregiver of the elderly as many of them were unemployed during the economic hardship of the pandemic period. No available formal training on the care of the elderly was obtained by any of the respondents before the module training. Therefore, we explored their experience during this critical period using the My-Elderly-Care-Skills module using existing guidelines and without providing any environmental support on technical components, such as internet access and access to a computer. It was challenging to fully understand the rigor required in qualitative research in view to replicate the thematic analysis as done in our study, especially when we conducted our research using an online platform. We could not appreciate in depth the emotions or feedback reflected because we did not have any contact with the respondents' surrounding environmental support.

## Conclusion

Exploration of the perceptions of the youth who have been trained to use the MY-Elderly-Care-Skills module in managing elderly care shows an important aspect of the technical and operational feasibility component. There were three themes obtained under operational feasibility, which were knowledge enhancement, the benefit of the care of the elderly, and improving awareness. The three themes for technical feasibility were the program completion in relation to digital communication barriers and the interactive approach. Future studies on applying digital education as a health promotion approach should focus on environmental support in the implementation of digital health education.

## Data availability statement

The raw data supporting the conclusions of this article will be made available by the authors, without undue reservation.

## Ethics statement

The studies involving human participants were reviewed and approved by UKM PPI/111/8/JEP-2021-754. The patients/participants provided their written informed consent to participate in this study.

## Author contributions

NM, RS, RMY, and HY participated and approved the study design. All researchers are women. NM, RS, and RMY contributed to designing the study. NM collected the data and conducted the FGD assisted by RS. NM and RS analyzed the data. NM, RS, and HY wrote the final article. All authors read and approved the final manuscript.
