# Supplementary material for: Youth perceptions toward managing elderly care among low-income household families using the My-Elderly-Care-Skills Module
Source: Front Public Health. 2023 Feb 8;11:1042124. doi: 10.3389/fpubh.2023.1042124 (PMC9945268; doi:10.3389/fpubh.2023.1042124)
Supplement: Supplementary file 1 [file Data_Sheet_1.docx]

Supplementary file 1


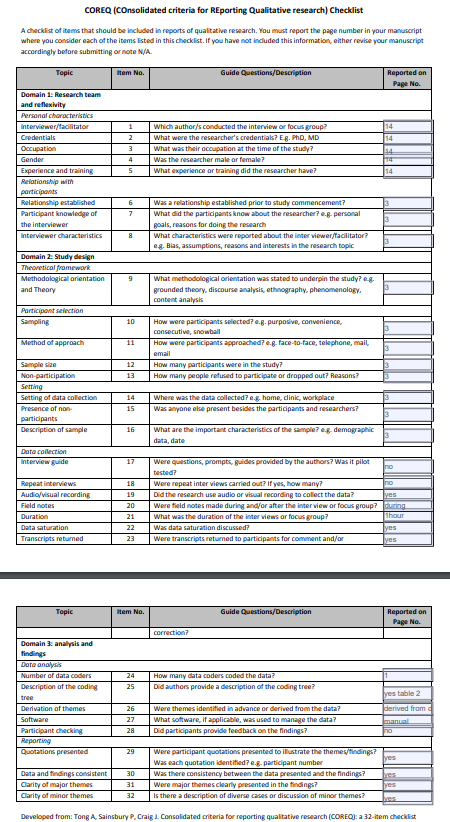


Supplementary file 2: **Division of respondents for FGD using the google meet application**

| Medium | Google Meet | |
| --- | --- | --- |
| **Links** | <https://meet.google.com/aqz-majz-ihf> | |
|  | | |
| **No** | **Name of Informant** | **Date and time** |
| 1. | Informant 1 | Monday  (4/10/21)  8:00 pm – 9:30 pm |
| 2. | Informant 2 |  |
| 3. | Informant 3 |  |
| 4. | Informant 4 |  |
| 5. | Informant 5 |  |
| 6. | Informant 6 | Tuesday  (5/10/21)  8:00 pm – 9:30 pm |
| 7. | Informant 7 |  |
| 8. | Informant 8 |  |
| 9. | Informant 9 |  |
| 10. | Informant 10 |  |
| 11. | Informant 11 | Wednesday  (6/10/21)  8:00 pm – 9:30 pm |
| 12. | Informant 12 |  |
| 13. | Informant 13 |  |
| 14. | Informant 14 |  |
| 15. | Informant 15 |  |
| 16. | Informant 16 | Thursday  (7/10/21)  8:00 pm – 9:30 pm |
| 17. | Informant 17 |  |
| 18. | Informant 18 |  |
| 19. | Informant 19 |  |
| 20. | Informant 20 |  |
| 21. | Informant 21 | Friday  (8/10/21)  8:00 pm – 9:30 pm |
| 22. | Informant 22 |  |
| 23. | Informant 23 |  |
| 24. | Informant 24 |  |
| 25. | Informant 25 |  |
| 26. | Informant 26 | Saturday  (9/10/21)  8:00 pm – 9:30 pm |
| 27. | Informant 27 |  |
| 28. | Informant 28 |  |
| 29. | Informant 29 |  |
| 30. | Informant 30 |  |

Supplementary file 3: **Key question asked during group discussion (FGD)**

| Part A: Introduction | Researcher questions and checklists in category scope  Thank you to all the participants of the My-Elderly-Care-Skills workshop who attended today and thank you for being a study participant and interviewed today. I am doing research related to 'Empowering B40 youth in the health management of the elderly on quality of life, social support and psychological well-being'. Therefore, the results of this interview will help me in completing my research.  (Introduce yourself / the purpose of the study and the context of elderly care researchers) |
| --- | --- |
| Part B: Background | As an introduction, participants are asked to explain a little background, including who is the elderly person who takes care of their parents/grandparents/where they live |
| Section C: Main questions/ content | 1. How do you find the My-Elderly-Care-Skills module and training in helping understanding and skills in improving healthcare for the elderly?  - Implications - Others - Description: (why)  1. What did you learn after attending the training webinar over the past two days ?  - Implications - Others - Description: (why)   3. Did this training webinar help you in caring for the elderly?   - Implications - Others - Description: (why)   4. What challenges do you face in using the information given in the  modules?   - Implications - Others - Description: (why) |
| Part D: Closing questions | Is there any additional point you would like to tell before we close?  Thank you |
